# Insights into the Current Management Techniques for Peri-Implant Gaps: A Systematic Review

**DOI:** 10.3390/jcm14103351

**Published:** 2025-05-12

**Authors:** Syed Kowsar Ahamed, Giovanni Battista Menchini-Fabris, Ali Alqarni, Shaimaa Mohammed Alarabi, Abdulaziz Abdullah Alharbi, Ammar Alshamrani, Ugo Covani, Saverio Cosola

**Affiliations:** 1 Department of Oral & Maxiloofacial Surgery and Diagnostic Sciences, Faculty of Dentistry, Taif University, Taif 21944, Saudi Arabia; 2 Department of Stomatology, Tuscan Stomatologic Institute, Foundation for Dental Clinic, Research and Continuing Education, 55041 Camaiore, Italycovani@covani.it (U.C.); 3 Department of Health Sciences, Pegaso University, 00187 Rome, Italy; 4 San Rossore Dental Unit, Casa di Cura San Rossore, 56122 Pisa, Italy; 5 Department of Restorative Dental Sciences, Faculty of Dentistry, Taif University, Taif 21944, Saudi Arabia; 6 Department of Dentistry, Unicamillus—Saint Camillus International University of Health and Medical Sciences, 00100 Rome, Italy

**Keywords:** dental implants, peri-implant gap, bone grafting, immediate implant placement, systematic review

## Abstract

**Objective:** A peri-implant gap or a “jumping gap” between an implant surface and the buccal bone can often complicate the successful integration of dental implants, impairing osseointegration and long-term implant stability. Although various techniques and materials are available for managing this gap, there is no consensus on the most effective approach. The current literature lacks standardized, evidence-based guidelines for selecting the optimal technique or material for managing peri-implant gaps, especially following immediate implant placement. This systematic review aims to evaluate the efficacy of various techniques and materials to manage the peri-implant gap to improve the implant stability, bone preservation, and esthetic outcomes using the PROSPERO registration number CRD42024508852. **Methods:** A comprehensive search of the MEDLINE, Embase, and Cochrane databases was conducted, and various studies were selected, including 11 randomized clinical trials that investigated different grafting materials and techniques for managing the gap between the implant and the buccal plate. The selected studies were assessed for the risk of bias, and the data were extracted based on primary outcomes such as implant stability, bone density, and esthetic parameters. **Results:** The findings indicate that xenografts and alloplastic grafts were superior in preserving bone volume compared to platelet-rich fibrin. Techniques like the socket shield and immediate provisional prothesis methods showed promise in maintaining soft tissue and bone integrity. However, heterogeneity across the studies limits definitive conclusions. **Conclusions:** Further high-quality research is needed to establish standardized guidelines for peri-implant gap management. The selection of techniques and materials should be tailored to individual patient needs.

## 1. Introduction

The successful integration of dental implants hinges on effectively managing the “jumping gap” or “jumping distance”, also known as the “peri-implant gap”, between the implant surface and the buccal bone plate following immediate implant placement. This critical space between the implant surface and the surrounding bone tissue presents a significant challenge in modern implant dentistry. This gap can challenge osseointegration and compromise long-term implant stability if not properly managed. This can lead to complications such as bone loss, peri-implantitis, and, ultimately, implant failure. Successful dental implant placement relies on achieving osseointegration. The peri-implant gap presents a challenge for osseointegration and compromises long-term implant stability. A more significant gap can compromise bone formation, leading to fibrous tissue encapsulation, bony defect formation, and compromised implant stability [1,2]. The effective management of this gap is essential to prevent complications like bone loss, peri-implantitis, and implant failure.

Managing the peri-implant gap effectively is fraught with challenges. One major challenge is the biological process of bone healing and regeneration, which can be unpredictable. Several challenges exist in managing the peri-implant gap. Bone healing after immediate implant placement is a complex biological process influenced by factors like the patient’s bone quality, infection, the size of the alveolar socket, buccal bone plate thickness, the procedure used, and implant stability [3,4]. The gap size plays a role, with more significant gaps requiring more sophisticated techniques for successful bone bridging [5].

Recent advancements have led to diverse techniques for managing the peri-implant gap. These techniques can be broadly categorized into surgical approaches, biomaterials, and innovative technological solutions. Surgical techniques enhance implant stability and promote bone regeneration within the jumping gap. One such technique is guided bone regeneration, which has been shown to prevent soft tissue infiltration and promote osseointegration in managing more significant jumping gaps [6,7]. Another surgical approach is the ridge augmentation technique, which aims to increase the bone volume and density for implant placement and stability. This is achieved using preferred autogenous bone grafts of bones harvested from the patients and grafted into the implant site or using allografts or xenografts [8,9].

As an advancement, the development and the use of biomaterials have revolutionized gap management. Bone graft materials like hydroxyapatite and tricalcium phosphate are commonly applied to fill the jumping gaps and promote bone regeneration. They provide scaffolds for new bone growth and are replaced by the patient’s bone by being resorbed over time [10,11]. In addition to those materials, bioactive implant surface coatings, such as bioactive glass or calcium phosphate, have been developed. These enhance osseointegration by enhancing the biological interaction between the implant and the nearby bone, thus improving the stability and longevity of the implant [12,13].

Technological advancements also contribute significantly to jumping gap management. The notable innovation of 3D printing allows for customized scaffolds and implants that precisely match the peri-implant gap, promoting targeted bone regeneration [14,15]. This technology also allows the incorporation of growth factors and other bioactive agents to enhance bone healing further. Another technological advancement using growth factors, like bone morphogenetic proteins and stem cells, can be applied to the implant site to stimulate new bone formation, enhance osteogenesis, and improve implant integration, thus promoting bone regeneration [16,17].

Immediate provisionalization, another technique in which a temporary restoration is placed on a dental implant concurrently with its insertion, has garnered increasing interest due to its potential advantages in peri-implant gap management. This approach may positively influence the healing process by creating a stable environment, promoting soft tissue healing, and enhancing esthetic outcomes. Additionally, immediate provisionalization can shape peri-implant soft tissues, potentially improving esthetics and achieving greater patient satisfaction [18].

Managing the dental jumping distance, or peri-implant gap, is crucial in implant dentistry to ensure successful osseointegration and long-term implant stability, as gaps can lead to bone loss and implant failure. Despite advancements in technology and materials, the effective management of this jumping gap remains challenging due to the variability in bone healing and regeneration processes. Therefore, a systematic review of the available literature addressing different techniques to manage the dental jumping gap is essential to evaluate and compare these approaches, providing evidence-based guidelines to enhance clinical practices and patient outcomes. Addressing this issue comprehensively will help standardize treatment protocols, improve implant success rates, and ultimately advance the field of implant dentistry.

This review aims to address the clinical question regarding the most effective technique for managing the dental jumping gap (peri-implant gap) between the implant and the buccal cortical bone following immediate implant placement to preserve the buccal bone level, gingival contour, and esthetics.

## 2. Methods

### 2.1. Ethics

This systematic review adheres to the Preferred Reporting Items for Systematic Review and Meta-Analysis Protocols 2015 (PRISMA-P) statement to ensure methodological rigor and transparency [19]. The protocol has been registered with the International Prospective Register of Systematic Reviews (PROSPERO) under registration number CRD42024508852.

### 2.2. Source

A thorough search strategy was employed to identify relevant studies, encompassing multiple electronic databases: MEDLINE (via PubMed), Embase, and the Cochrane Central Register of Controlled Trials. This search included publications from January 2013 to the present because in the last 15 years some studies investigating alternatives to the use of biomaterials have been published, such as immediate protheses or socket shields.

To supplement database searching, Google Scholar was utilized to identify the potentially relevant grey literature, including conference abstracts and dissertations. Additionally, reference lists of all the included studies and pertinent systematic reviews underwent manual screening to ensure the comprehensive retrieval of eligible articles. The search was limited to English-language publications.

### 2.3. Search Strategy

A comprehensive database of published studies investigating the effectiveness of various techniques for managing implant jumping gaps following immediate implant placement was compiled. This involved a rigorous search strategy across the mentioned databases, employing a combination of MeSH terms, free-text terms, and Boolean operators (“OR”, “AND”, “NOT”) to maximize the search sensitivity and specificity, as reported in Table 1, following the PRISMA guidelines. The search encompassed the following key concepts: “immediate implant placement”, “implant jumping gap”, “jumping distance”, “peri-implant gap”, “gap management”, “bone grafting”, “guided bone regeneration”, “connective tissue graft”, “provisionalization”, “soft tissue contour”, “buccal bone level”, “implant stability”, and “esthetics”. (Table 1). The search strategy was tailored to each database’s specific syntax and features to ensure extensive retrieval. After the 7 steps (#7) had been followed, the 30 results were analyzed.

### 2.4. Eligibility Criteria

Studies were considered eligible for inclusion based on the following PICOS (participants, interventions, Comparators, outcomes, and study design) criteria:

#### 2.4.1. Participants

Studies involving human participants of any age requiring immediate implant placement with a jumping distance or peri-implant gap were considered.

#### 2.4.2. Interventions

Studies evaluating any technique for managing the jumping distance or peri-implant gap, including but not limited to bone grafting, guided bone regeneration, soft tissue augmentation, and/or immediate provisionalization, were eligible.

#### 2.4.3. Comparators

Studies were included that compared different interventions for managing the jumping distance/peri-implant gap or compared an intervention to a control group (e.g., no intervention, delayed implant placement).

#### 2.4.4. Outcomes

Studies reporting on at least one of the following primary outcomes were considered: changes in soft tissue contour, buccal bone levels, or esthetic outcomes. Secondary outcomes of interest included implant stability and implant survival rates.

#### 2.4.5. Study Design

Randomized controlled trials, randomized clinical trials, cohort studies, and case–control studies investigating the management of the jumping distance or peri-implant gap in immediate implant placement were included.

#### 2.4.6. Language

Only studies published in English were included.

#### 2.4.7. Study Location

Studies were included without considering any geographical exclusion criteria.

#### 2.4.8. Publication Year

2013–2024

Studies were excluded if they were case reports, case series with fewer than 10 patients, reviews, editorials, or commentaries, focused solely on immediate implant placement without addressing the jumping distance or peri-implant gap management, or did not report on any of the pre-specified primary or secondary outcomes.

### 2.5. Study Selection

Following the initial search, all the identified citations were imported into the Rayyan reference management software, and duplicate citations were removed. Two reviewers independently screened the titles and abstracts of the remaining citations to identify potentially eligible studies based on the predefined eligibility criteria. Both reviewers retrieved and assessed full-text articles of these potentially eligible studies in detail. Any disagreements between the reviewers regarding the eligibility of a study were resolved through discussion and consensus or by consulting a third reviewer if needed.

### 2.6. Data Extraction and Management

A standardized data extraction form was developed and piloted to ensure consistency in data collection. Two reviewers independently extracted data from the included studies using this form. The extracted data encompassed the following:

#### 2.6.1. Study Characteristics

The author, year of publication, study design, sample size, participant demographics, intervention details, and other relevant study design elements.

#### 2.6.2. Outcome Data

Mean values with standard deviations or medians with interquartile ranges for continuous outcomes and frequencies and proportions for categorical outcomes are reported at baseline and various follow-up time points.

#### 2.6.3. Risk-of-Bias Assessment

An assessment of the risk of bias for each study was performed using the chosen tools: RoB 2 for randomized trials or ROBINS-I for non-randomized studies.

Disagreements that arose during the data extraction process were resolved through discussion between the two reviewers to achieve a consensus. If necessary, a third reviewer was consulted to resolve persistent discrepancies.

## 3. Results

**Figure 1:**
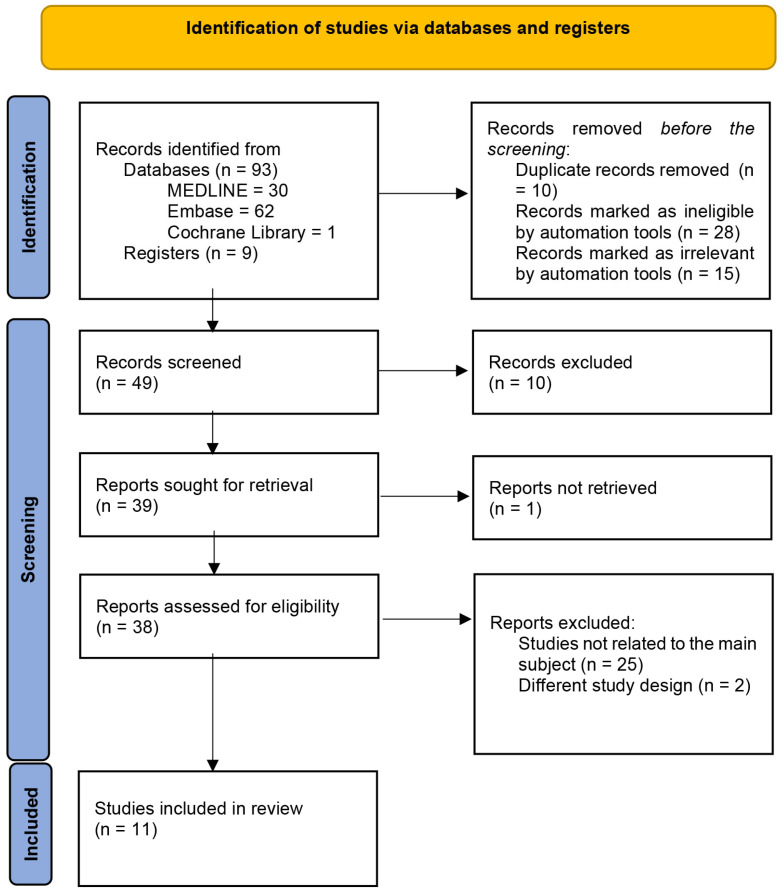
PRISMA Flow diagram of identification and inclusion of papers [19].

The PRISMA flow diagram illustrates the study selection process (Figure 1).

After a comprehensive search of the literature was conducted across multiple databases to identify potentially relevant studies, an initial yield of 102 records was subsequently reduced to 49 by removing duplicates and applying rigorous eligibility criteria. A total of 10 records were excluded because the words “peri-implant gap” and “jumping gap” were not included in the title or abstract. The full-text articles were obtained for 39 of these records, with 1 article proving inaccessible. A detailed assessment excluded 27 studies due to irrelevance or inappropriate study design, leaving 11 studies eligible for inclusion in the final review (Table 2).

These selected studies were randomized controlled trials evaluating various grafting materials and their impacts on peri-implant outcomes.

El Ebiary et al. (2023) reported that grafting the gap between the implant and the bone with a combination of xenografts and autogenous bone may result in significantly higher esthetic outcomes after six months compared to conventional implant placement without any grafting (*p* = 0.048) [19].

Samy et al. (2024) found that the use of a mineralized plasmatic matrix reduced marginal bone loss and improved bone density compared to β-tricalcium phosphate, although the differences in implant stability were not significant [20].

Elsheikh et al. (2023) demonstrated that xenografts and alloplastic materials better preserved the buccal bone compared to platelet-rich fibrin (*p* < 0.001), while implant stability and peri-implant pocket depths showed no significant differences [21].

Amer et al. (2022) showed that filling materials containing hyaluronic acid and melatonin significantly improved gingival indices and reduced the probing depth after six months (*p* < 0.05 and *p* = 0.001, respectively) [22].

Elbrashy et al. (2022) highlighted that bovine cancellous xenografts significantly maintained crestal bone levels compared to platelet-rich fibrin (*p* = 0.002) [23].

Hammad et al. (2021) observed minimal marginal bone loss with both mixed allograft–xenograft and xenograft alone, with no significant differences in esthetic scores [24].

Shaaban Metwally et al. (2021) found that platelet-rich fibrin significantly enhanced the bone density around implants over nine months (*p* = 0.039) [25].

Atef et al. (2021) reported that the socket shield technique better preserved midfacial mucosal tissues compared to bovine xenografts (*p* = 0.017), without compromising esthetic outcomes [26].

Adam et al. (2020) demonstrated that NanoBone grafts maintained higher marginal bone levels than autogenous chin bone grafts, despite demonstrating similar implant stability [27].

Naji et al. (2019) concluded that both the “flap with graft” and “flapless without graft” techniques provided similar horizontal bone healing in premolar sites [28].

Finally, Sanz et al. (2016) revealed that using deproteinized bovine bone mineral with collagen (DBBM-C) significantly reduced horizontal crest resorption compared to no grafting (*p* = 0.018–0.029) [29].

### 3.1. Risk-of-Bias Analysis

Given that the 11 included studies were of “Randomized Clinical Trial” or “Randomized Control Trial” design, the risk-of-bias assessment was conducted using the “Cochrane Risk of Bias tool (RoB 2)”. The evaluation focused on domains including randomization, deviations from the intended intervention, missing outcome data, outcome measurements, and reporting biases. While most of the studies exhibited a low risk of bias across these domains, “specific concerns” were identified in certain studies about randomization and deviations from the intended interventions.

**Figure 2:**
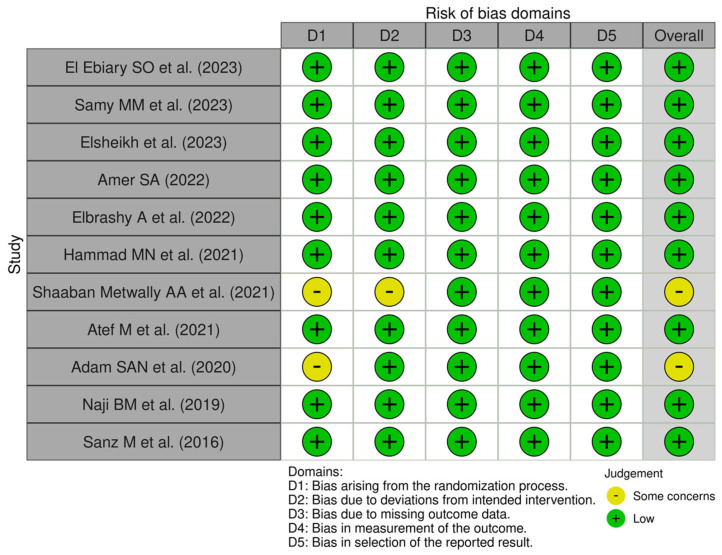
Traffic Light Plot for the included studies [19,20,21,22,23,24,25,26,27,28,29].

**Figure 3:**
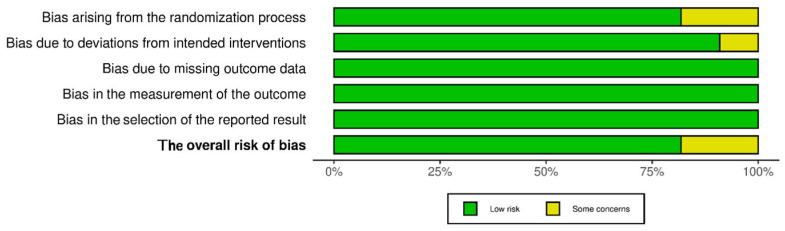
Summary Plot of the included studies.

In the study conducted by Shaaban Metwally AA et al. (2021), despite being labeled as a randomized clinical trial, the methodology lacked explicit details regarding randomization procedures, allocation concealment, and blinding techniques for the participants and caregivers [25]. Furthermore, the study did not address potential deviations from the intended intervention. Similarly, the study by Adam SAN et al. (2020) exhibited significant differences in the baseline data for outcome measures, suggesting potential heterogeneity between groups that could have influenced the study results [27]. Biases arising from the randomization process can potentially influence participant enrollment, leading to an overestimation of the study results. Additionally, deviations from the intended intervention may exaggerate the observed intervention effect compared to studies that adhere to standard approaches. Detailed analyses are demonstrated in Figure 2 and Figure 3.

### 3.2. The Characteristics and Outcomes of the Included Studies

All the studies adhered to a “Randomized Controlled Trial” or “Randomized Clinical Trial” design. The follow-up periods ranged from six to eighteen months. The participants were male and female patients presenting with non-restorable teeth requiring immediate implant placement, primarily in the maxillary arch.

The included studies exhibit a degree of heterogeneity due to variations in the study populations, outcome measures, and methodological approaches. These differences may influence the comparability of results and limit the generalizability of findings. While most of the studies included adults, the specific age ranges varied, potentially influencing the outcomes. Younger individuals may have different healing characteristics compared to older adults. The proportion of males and females varied across the studies, which could impact the results due to potential gender-specific differences in bone density and healing. Hormonal factors and anatomical variations between males and females may influence implant success. The studies included patients with non-restorable upper anterior teeth, maxillary premolars, and molars, suggesting variations in the anatomical challenges and treatment requirements. The implant placement was indicated in the esthetic zone (anterior maxilla) for some studies, while others focused on the premolar region. This variation could influence the complexity of the surgical procedure and the esthetic demands.

Across the studies, various intervention groups were employed, involving comparisons between different bone grafting materials, such as xenografts, allografts, autogenous bone, platelet-rich fibrin, and control groups without grafting. Primary outcome measures encompassed implant stability, bone density, marginal bone loss, esthetic outcomes, and soft tissue changes. The assessment of outcome measures varied across the studies, potentially influencing the overall evaluation of implant success. The inconsistency in outcome measures makes it challenging to directly compare the results across the different studies.

## 4. Discussion

This review analyzed the effects of different grafting materials and techniques on implant stability, marginal bone preservation, soft tissue outcomes, and esthetic results following immediate implant placement. The findings across the included randomized controlled trials showed significant variability depending on the material used and the clinical outcome assessed, and based on the included research papers, it is evident that the choice of materials significantly impacts the clinical outcomes. Regarding the esthetic outcomes, El Ebiary et al. demonstrated that grafting the jumping gap with a combination of xenografts and autogenous bone significantly improved the pink esthetic scores compared to conventional implant placement without grafting [19]. Similarly, Atef et al. reported that the socket shield technique better preserved the midfacial mucosal architecture without negatively affecting the esthetic appearance, highlighting the importance of gap management in maintaining soft tissue contours [26].

In terms of marginal bone preservation, Samy et al. [20] showed that a mineralized plasmatic matrix reduced marginal bone loss more effectively than β-tricalcium phosphate. Elbrashy et al. [23] also observed significantly less crestal bone loss and buccopalatal dimensional changes when using bovine cancellous xenografts compared to PRF, emphasizing the superiority of certain bone substitutes in maintaining hard tissue volume.

For implant stability, most studies (Samy et al., Elsheikh et al., Hammad et al., and Adam et al.) reported no significant differences between different grafting materials, suggesting that primary implant stability is more influenced by the surgical technique and the implant design, rather than by the choice of graft material [20,21,24,27].

The bone density outcomes varied. Shaaban Metwally et al. found that platelet-rich fibrin significantly enhanced the bone density over time compared to non-PRF-treated sites, while Samy et al. observed better preoperative bone density when using a mineralized plasmatic matrix compared to β-tricalcium phosphate [20,25].

In terms of soft tissue health, Amer et al. demonstrated that filling the jumping gap with hyaluronic acid and melatonin gels improved gingival indices and reduced probing depths significantly compared to no filling material [22]. These findings suggest that bioactive gels may play a supportive role in enhancing peri-implant soft tissue healing.

The findings from Sanz et al. reinforced the role of grafting materials, such as DBBM-C, in minimizing horizontal crest resorption, particularly in cases with thin buccal bone walls [29].

Despite promising results, certain studies showed no significant differences in clinical outcomes between materials, such as Hammad et al. for marginal bone loss and pink esthetic scores and Adam et al. for implant stability, suggesting that while grafting materials can provide benefits, their impact may sometimes be limited by patient-related factors or surgical protocols [24,27].

Overall, the evidence suggests that the choice of grafting material significantly influences hard and soft tissue outcomes following immediate implant placement. Xenografts, alloplasts, and biologically active materials, like PRF and hyaluronic acid/melatonin gels, seem particularly effective in enhancing esthetic and biological results. However, the heterogeneity in study designs, sample sizes, and follow-up durations warrants caution in generalizing these findings. Future long-term, multicenter, randomized controlled trials are necessary to confirm these results and optimize treatment protocols.

Among the grafting materials, xenograft and alloplastic bone grafts demonstrated superior outcomes in preserving buccal bone thickness, minimizing bone loss, and having relatively better implant stability as compared to platelet-rich fibrin (PRF) and autogenous bone, as evidenced by the mean buccal bone loss and implant stability quotient score in the PRF groups [21,23,24,26,28]. A similar result was demonstrated when a comparative analysis of PRF and cancellous bovine bone in immediate implant placement revealed a reduced mean bone loss and improved implant stability with xenograft utilization [29]. However, another study demonstrated contrary results while comparing PRF with implants without PRF, which has been shown to increase bone density around newly placed implants, thus suggesting the efficiency of PRF in stimulating bone growth and improving the overall stability of the implant [25].

Meanwhile, the evaluation of pink esthetic scores (PESs) in the anterior maxilla demonstrated enhanced esthetic outcomes as well, following the grafting of the jumping gap with 50% xenografts and 50% autogenous grafts, as suggested by a higher mean PES observed at the six-month mark [19]. However, a study evaluating immediate provisionalization using a mixture of allograft and xenograft versus xenograft alone found comparable effectiveness in preserving marginal bone and soft tissue esthetics [24]. Xenografts and allografts have been reported to augment the osseous volume and prolong osseointegration during re-ossification [30,31].

Deproteinized bovine bone, a mineralized plasmatic matrix (MPM), β-tricalcium phosphate (β-TCP), 1.2% topical hyaluronic acid gel, and melatonin gel were among the other materials used for the jumping gap fillings within the included studies. Using deproteinized bovine bone mineral with a 10% collagen filling as the filling material in conjunction with immediate implants significantly reduced the mean horizontal bone resorption [29]. Additionally, a melatonin gel and hyaluronic acid application improved soft tissue health and minimized the mean buccal bone resorption [22]. In the mandibular molar region, MPM outperformed β-TCP regarding implant stability, bone density, and marginal bone preservation, as indicated by the larger effect sizes for MPM in these parameters [20]. A NanoBone synthetic bone graft, composed of nanostructured hydroxyapatite particles embedded within a silica gel matrix, was compared to an autogenous graft and resulted in a similarly stable implant but caused less harm than the latter [30].

On the other hand, while considering the grafting process, comparing soft and hard tissue dimensional changes around immediate implants using the socket shield technique versus a xenograft demonstrated superior bone preservation with the socket shield technique [31]. The success of the socket shield technique may be traced back to its capacity to preserve the integrity and vascular supply of the root–periodontal connective tissue and to reduce the mechanical contraction of the buccal bone, thereby preventing anticipated post-extraction bone resorption and providing support for the buccal peri-implant tissues [32]. Moreover, comparing three methods of placing implants—with or without an alloplastic nanocrystalline calcium sulphate bone graft in the presence of a mucoperiosteal flap—the researchers found that all the methods resulted in similar bone preservation around the implant. This suggests that the presence or absence of a flap might not significantly affect bone healing in certain situations.

Although various grafting materials were evaluated, it is notable that allogenic bone grafts were not extensively studied among the included trials. Only Hammad et al. assessed a mixture of allograft and xenograft materials, finding minimal differences in the marginal bone loss and esthetic outcomes compared to a xenograft alone [24]. This limited evidence suggests that the specific impact of allogenic grafts on immediate implant outcomes remains unclear and warrants further investigation in future studies.

Thus, this review’s findings suggest that the careful selection of grafting materials and techniques can significantly impact the outcomes of immediate implant placement. Using xenografts and alloplastic bone grafts, as well as the socket shield technique, may be beneficial in preserving bone and enhancing esthetic outcomes [32]. However, further research is needed to establish the optimal treatment protocols.

Heterogeneity and the risk of bias significantly limited the ability to draw definitive conclusions from the included studies. Variation among the included studies, including the patient populations and outcome measures, hindered the pooling of the data for the analysis. Additionally, the risk of bias in some studies, particularly related to randomization and missing outcome data, may have influenced the results.

However, due to the high heterogeneity in study designs, populations, and outcome measures among the included trials, applying a “grade” framework was deemed inappropriate as it could have led to misleading conclusions.

Moreover, the conventional extraction surgery consisted of using elevators and forceps, which could easily damage the coronal aspect of the buccal and palatal/lingual cortical bone of the alveolar crest, thereby influencing the jumping gap [33].

Different grafting materials, exogenous or alloplastic bone substitutes, and various techniques were explored in this review; however, some clinical retrospective studies and animal studies that were not included highlighted that bone volume may be influenced by managing the tissues surrounding teeth. Implants also influence bone volume because the natural closure of the mound of soft tissue during initial healing makes apoptosis or the non-activation of myofibroblasts play a role in bone contraction [34,35].

This analysis identified several methods to manage the jumping gap and to reduce the bone resorption in the first months after a post-extractive implant is placed, and these methods consist of two main considerations:

1. The mechanical support of the periosteum: by filling the alveolus with biomaterials, the mechanical support may be performed by socket shield techniques, which result in the minimum bone loss or the best bone volume maintenance [32,36];
2. The inhibition of myofibroblast activities (especially in the first three weeks of soft tissue healing). This can be achieved via the fast, complete wound epithelialization of the socket by using the following:
3. An immediate provisional prothesis (or customized healing screws) to close the gap between the alveolus and the free gingiva [35,37,38,39,40,41,42];
4. A free gingival graft to close the gap between the two portions of the flap that was designed during the implant surgery [43];
5. Platelet concentrations with the aim of closing the gap between the two portions of the flap designed during the implant surgery and filling it in with a natural biomaterial [44].

These clinical retrospective studies seem to highlight that an immediate provisional prothesis could maintain the esthetic outcomes and the thickness and shapes of the gingival parabola and could reduce the bone volume loss. In view of residual bone preservation immediately after the extraction, all surgical techniques with different technologies (magnetic mallet, bone condensation, piezo-surgery) that involve immediate implant placement and an immediate provisional prothesis are proposed as being Prosthetic Socket Preservation (PSP) techniques.

To address the heterogeneity of the studies included, as well as the further high-quality randomized clinical/controlled trials with larger sample sizes that were not included, standardized outcome measures and detailed review summarizations are required to strengthen the evidence base. Moreover, long-term follow-up studies are essential to assess the durability of treatment effects and potential complications.

## 5. Conclusions

The present systematic review underscores the pivotal role of grafting materials and techniques in achieving optimal outcomes for managing peri-implant gaps or jumping gap distance in patients undergoing immediate implant placement. While xenografts and alloplastic bone grafts demonstrated superior performance in preserving bone and enhancing implant stability, the efficacy of PRF in specific contexts warrants further investigation. The potential benefits of combined therapies, such as PRF and bone grafts, also merit exploration. The evaluation of immediate provisional prothesis or customized abutment to manage the peri-implant gap by using a prothesis could have advantages in the bone volume and esthetic outcomes by promoting soft tissue healing.

Ultimately, the selection of grafting materials and techniques should be individualized based on patient-specific factors and clinical judgment.

## Figures and Tables

**Table 1:** MEDLINE search strategy.

Search Number	Query	Results
#7	((((soft tissue contour) OR (buccal bone level)) OR (implant stability)) OR (esthetics)) AND #6	30
#6	#4 AND #5	46
#5	(((bone grafting) OR (guided bone regeneration)) OR (connective tissue graft)) OR (provisionalization)	90,733
#4	((immediate implant placement) AND (((implant jumping gap) OR (jumping distance)) OR (peri-implant gap)) AND ((gap management) AND (2013:2024[pdat]))	11
#3	((immediate implant placement) AND (2013:2024[pdat])) AND (((implant jumping gap) OR (jumping distance)) OR (peri-implant gap) AND (2013:2024[pdat]))	220
#2	((implant jumping gap) OR (jumping distance)) OR (peri-implant gap)	1437
#1	(immediate implant placement)	5851

**Table 2:** The descriptive characteristics of the included studies.

Study	Method	Participants	Interventions (*n*)	Outcomes	Results	Conclusion
El Ebiary SO et al. (2023) [19]	RCliT (6 months)	24 (9 males, 15 females) patients were indicated for extraction and immediate implant installation therapy with non-restorable upper anterior teeth in the esthetic zone.	Group 1: bone grafts with 50% xenografts and 50% autogenous grafts (12). Group 2: conventional implant without grafting (12).	Pink esthetic score (immediately post-intervention, 6 months).	Baseline: Study: 11.58 (1.16). Control: 11.75 (1.71). *p*-value: 0.746. After 6 months: Study: 12.42 (1.44). Control: 11.17 (1.53). *p*-value: 0.048.	Grafting the jumping gap results in higher esthetic outcomes in the anterior maxilla.
Samy MM et al. (2023) [20]	RCliT (6 months)	16 patients indicated for immediate dental implant following badly decayed molars.	Group 1: mineralized plasmatic matrix as graft (8). Group 2: β-tricalcium phosphate as graft (8).	Implant stability. Marginal bone loss. Bone density (6 months).	Implant stability immediately post-operation: Group 1: 58. Group 2: 62.5. *p*-value: 0.224. After 6 months: Group 1: 70.5. Group 2: 70. *p*-value: 0.557. Marginal bone loss: Group 1: 0.3. Group 2: 0.35. *p*-value: 0.040. Pre-operative bone density: Group 1: 632.5. Group 2: 735. *p*-value: 0.040. Immediately post-operation: Group 1: 637.5. Group 2: 745. *p*-value: 0.058. After 6 months: Group 1: 850. Group 2: 815. *p*-value: 0.635.	A mineralized plasmatic matrix promotes better bone growth and implant stability in the mandibular region than β-tricalcium phosphate as a graft.
Elsheikh HA et al. (2023) [21]	RCliT (18 months)	36 patients (19 females and 17 males) seeking immediate implant replacement for non-restorable maxillary anterior and premolar teeth in the esthetic zone.	Group 1: platelet-rich fibrin placed into the jumping gap (12). Group 2: xenograft (12). Group 3: alloplastic β-tricalcium phosphate bone grafting (12).	Implant stability. Peri-implant pocket depth. Buccal bone changes.	Implant stability at surgery: Group 1: 64.33 (2.77). Group 2: 65.08 (2.27). Group 3: 66.33 (2.57). *p*-value: 0.114. After 6 months: Group 1: 71.83 (2.41). Group 2: 72.35 (2.35). Group 3: 73.83 (3.16). *p*-value: 0.119. After 18 months: Group 1: 73.50 (2.07). Group 2: 73.92 (2.39). Group 3: 74.92 (1.38). *p*-value: 0.216. Peri-implant pocket depth after 6 months: Group 1: 1.54 (0.23). Group 2: 1.40 (0.25). Group 3: 1.57 (0.43). *p*-value: 0.396. After 18 months: Group 1: 2.42 (0.46). Group 2: 2.32 (0.33). Group 3: 2.49 (0.27). *p*-value: 0.533. Buccal bone changes: Group 1: 1.56 (0.52). Group 2: 0.65 (0.31). Group 3: 0.69 (0.32). *p*-value: <0.001.	Xenograft and alloplastic bone grafts better preserve the bone around immediate implants than platelet-rich fibrin.
Amer SA (2022) [22]	RCliT (6 months)	32 patients needing immediate implants in the premolar region of the maxillary teeth.	Group 1: no filling material (8). Group 2: 1.2% topical hyaluronic acid gel as a filling material (8). Group 3: 1.2% hyaluronic acid gel plus melatonin gel as a filling material (8). Group 4: melatonin gel as a filling material (8).	Clinical evaluation. Gingival index. Probing depth (immediately post-intervention, 6 months).	Baseline gingival index: Group 1: 1.65 (0.37). Group 2: 1.70 (0.27). Group 3: 1.70 (0.38). Group 4: 1.74 (0.29). *p*-value: 0.889. Probing depth: Group 1: 3.71 (0.61). Group 2: 3.64 (0.67). Group 3: 3.73 (0.64). Group 4: 3.79 (0.63). *p*-value: 0.949. Gingival index after 6 months: Group 1: 1.23 (0.69). Group 2: 1 (0.52). Group 3: 0.35 (0.54). Group 4: 0.5 (0.55). *p*-value: <0.05. Probing depth: Group 1: 3.54 (0.41). Group 2: 3.61 (0.31). Group 3: 2.67 (0.24). Group 4: 2.61 (0.21). *p*-value: 0.001. (Group C,D)	Neither melatonin gel nor hyaluronic acid prevented bone loss around the implants.
Elbrashy A et al. (2022) [23]	RCliT (6 months)	20 patients (11 males and 9 females) seeking immediate implant replacement, suffering from non-restorable maxillary premolars.	Group 1: bovine cancellous xenograft (10). Group 2: platelet-rich fibrin to graft (10).	Crestal bone loss. Buccopalatal dimensions change. Implant stability. Pink esthetic zone.	Crestal bone loss: Study: 1.85 (0.89). Control: 0.77 (0.32). *p*-value: 0.002. Buccopalatal dimensions change: Study: 1.63. Control: 0.59. *p*-value: <0.001. Implant stability: Study: 74 (9.0). Control: 64 (9.0). *p*-value: 0.023. Pink esthetic zone: Study: 10.9 (1.52). Control: 11.9 (1.60). *p*-value: 0.169.	A xenograft as a gap distance-filling material significantly maintained the crestal bone level surrounding the implant.
Hammad MN et al. (2021) [24]	RCliT (6 months)	17 (7 males and 10 females) patients indicated for immediate implant placement with non-restorable maxillary teeth on 20 extraction sockets.	Group 1: mixture of allograft and xenograft (10 extractions in 9 patients). Group 2: xenograft (10 extractions in 8 patients).	Marginal bone loss (6 months). Pink esthetic score (immediately post-intervention, 6 months).	Marginal bone loss: Group 1: 0.43 (0.2). Group 2: 0.34 (0.1). *p*-value: 0.219. Pink esthetic zone at baseline: Study: 12.4 (1.07). Control: 12.7 (0.82). *p*-value: 0.49. After 6 months: Study: 11.7 (1.05). Control: 12.1 (10.73). *p*-value: 0.336.	Both bone graft types showed minimal bone loss, slightly more in the mixed graft group, and a similar gum appearance after implant placement.
Shaaban Metwally AA et al. (2021) [25]	RCliT (9 months)	Patients with unrestorable teeth, indicated for implant placement.	Group 1: platelet-rich fibrin. Group 2: without protein-rich fibrin.	Bone density (immediately post-intervention, 3 months, 6 months, 9 months).	Baseline: Study: 572.77 (33.29). Control: 568.38 (47.18). *p*-value: 0.413. After 3 months: Study: 712.12 (32.70). Control: 663.97 (34.12). *p*-value: 0.136. After 6 months: Study: 979.57 (82.86). Control: 800.05 (53.88). *p*-value: 0.732. After 9 months: Study: 1139.2 (65.51). Control: 972.45 (64.18). *p*-value: 0.039.	Protein-rich fibrin effectively enhances bone density around immediate implants placed in the esthetic zone.
Atef M et al. (2021) [26]	RCliT (12 months)	42 patients (12 males and 30 females), each with a single non-restorable tooth in the esthetic zone to be replaced with an immediate implant.	Group 1: ungrafted socket shield method (21). Group 2: bovine cancellous xenograft (21).	Esthetic outcomes. Pink esthetic score. Midfacial mucosal alteration (12 months).	Pink esthetic score: Study: 12.2 (0.64). Control: 11.86 (0.35). *p*-value: 0.333. Midfacial mucosal alteration: Study: 0.45 (0.75). Control: −0.47 (0.58). *p*-value: 0.017.	The socket shield method preserved bone and soft tissue better than traditional grafting after immediate implant placement without affecting gum appearance or patient satisfaction.
Adam SAN et al. (2020) [27]	RCT (6 months)	18 (8 males, 10 females) patients were indicated for immediate implant placement with an unrestorable single tooth.	Group 1: NanoBone grafts (9). Group 2: autogenous bone from the chin (9).	Marginal bone level (immediate post-intervention, 6 months). Implant stability quotient (6 months).	Marginal bone level at baseline: Mesial: Group 1: 1.42 (0.45). Group 2: 0.97 (0.06). *p*-value: 0.009. 2. Distal: Group 1: 1.49 (0.36). Group 2: 0.88 (0.07). *p*-value: 0.0001. After 6 months: Mesial: Group 1: 0.71 (0.08). Group 2: 0.53 (0.06). *p*-value: 0.0001. 2. Distal: Group 1: 0.79 (0.08). Group 2: 0.32 (0.06). *p*-value: 0.0001. Implant stability quotient: Average: Group 1: 62.44 (6.86). Group 2: 63.36 (10.91). *p*-value: 0.833.	Both methods resulted in similarly stable implants, but NanoBone caused less harm to the patients.
Naji BM et al. (2019) [28]	RCliT (6 months)	48 patients (18 males and 30 females) were indicated for dental implant placement for unrestorable maxillary premolar.	Group 1: mucoperiosteal flap with alloplastic nanocrystalline calcium sulfate bone graft (16). Group 2: flap without graft (16). Group 3: flapless without graft (16).	The horizontal dimension of the buccal alveolar bone (immediately post-intervention, 6 months).	Baseline–6 months: Group 1: 0.37 (0.09). Group 2: 0.91 (0.54). Group 3: 0.24 (0.11). *p*-value: 0.003.	Both “flapless without graft” and “flap with graft” showed similar bone healing after implant placement in premolars with adequate bone thickness.
Sanz M et al. (2016) [29]	RCliT (16 weeks)	86 (41 males and 45 females) patients who needed at least one tooth in the anterior maxilla to be removed and replaced with implants.	Group 1: deproteinized bovine bone mineral with 10% collagen (DBBM-C) filling (43). Group 2: no graft (43).	Crest dimensions. Thin buccal site (≤1 mm). Anterior site. Sites with a gap size of ≥2 mm (immediately post-intervention, 6 months).	Baseline–4 months: Buccolingual dimension: Study: −2.19 (2.10). Control: −2.65 (1.81). *p*-value: 0.149. Alveolar crest width: Study: −1.26 (1.75). Control: 1.71 (1.36). *p*-value: 0.187. Horizontal crest dimension: Study: −1.07 (1.10). Control: 1.59 (1.05). *p*-value: 0.029. Horizontal gap dimension: Study: −1.57 (−1.27). Control: −2.23 (1.22). *p*-value: 0.018. Vertical defect dimension: Study: −6.97 (2.68). Control: −6.45 (3.24). *p*-value: 0.43.	Using DBBM-C bone graft helped prevent horizontal bone resorption around newly placed implants.
